# Dynamic Finite Size Effects in Spiking Neural Networks

**DOI:** 10.1371/journal.pcbi.1002872

**Published:** 2013-01-24

**Authors:** Michael A. Buice, Carson C. Chow

**Affiliations:** Laboratory of Biological Modeling, NIDDK, NIH, Bethesda, Maryland, United States of America; University of Pittsburgh, United States of America

## Abstract

We investigate the dynamics of a deterministic finite-sized network of synaptically coupled spiking neurons and present a formalism for computing the network statistics in a perturbative expansion. The small parameter for the expansion is the inverse number of neurons in the network. The network dynamics are fully characterized by a neuron population density that obeys a conservation law analogous to the Klimontovich equation in the kinetic theory of plasmas. The Klimontovich equation does not possess well-behaved solutions but can be recast in terms of a coupled system of well-behaved moment equations, known as a moment hierarchy. The moment hierarchy is impossible to solve but in the mean field limit of an infinite number of neurons, it reduces to a single well-behaved conservation law for the mean neuron density. For a large but finite system, the moment hierarchy can be truncated perturbatively with the inverse system size as a small parameter but the resulting set of reduced moment equations that are still very difficult to solve. However, the entire moment hierarchy can also be re-expressed in terms of a functional probability distribution of the neuron density. The moments can then be computed perturbatively using methods from statistical field theory. Here we derive the complete mean field theory and the lowest order second moment corrections for physiologically relevant quantities. Although we focus on finite-size corrections, our method can be used to compute perturbative expansions in any parameter.

## Author Summary

One avenue towards understanding how the brain functions is to create computational and mathematical models. However, a human brain has on the order of a hundred billion neurons with a quadrillion synaptic connections. Each neuron is a complex cell comprised of multiple compartments hosting a myriad of ions, proteins and other molecules. Even if computing power continues to increase exponentially, directly simulating all the processes in the brain on a computer is not feasible in the foreseeable future and even if this could be achieved, the resulting simulation may be no simpler to understand than the brain itself. Hence, the need for more tractable models. Historically, systems with many interacting bodies are easier to understand in the two opposite limits of a small number or an infinite number of elements and most of the theoretical efforts in understanding neural networks have been devoted to these two limits. There has been relatively little effort directed to the very relevant but difficult regime of large but finite networks. In this paper, we introduce a new formalism that borrows from the methods of many-body statistical physics to analyze finite size effects in spiking neural networks.

## Introduction

Realistic models of neural networks in the central nervous system are analytically and computationally intractable, presenting a challenge to our understanding of the highly complex spiking dynamics of neurons. Consequently, some degree of simplification is necessary for theoretical progress and there is a corresponding spectrum of models with a range of complexity. “Mean Field” models represent the highest degree of simplification and classically consider the evolution of an “activity” variable which is some average of the output of a population of neurons. Early examples of mean field models are those of Wilson-Cowan [1], [2], Cohen and Grossberg [3], and Amari [4]. These models have proven to be useful in studies of neural dynamics such as in pattern formation and visual hallucinations [5]–[7]. However, because of the nature of the activity variables as averages, they necessarily neglect individual neuron dynamics as well as population level effects of phase information and synchrony. Additionally, it is not clear how the time scales in the equations of mean field models are related to the response properties of the constituent neurons [8].

The next level of model complexity requires relating population level activity to single neuron dynamics. This is a question explored by Knight [9], [10], who noted in particular that although the population firing rate may track an external stimulus, the single neuron firing rate need not and generally does not. The important conceptual feature introduced was that a population of neurons, each of which has some potential variable, 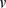, can be replaced with a density, 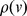, which counts the fraction of neurons whose potential lies within the infinitesimal range 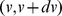. The firing rate of the population is then the current density of the population at the threshold potential. In the limit of an infinite population of neurons, one can introduce a continuity equation derived from the single neuron dynamics, producing what can be called *density mean field theory*. The density mean field approach to analyzing coupled networks has been pursued by Desai and Zwanzig [11], Strogatz and Mirollo [12]–[14], Treves [15], Abbott and van Vreeswijk [16] and others [17]–[26]. The spike response formalism considers an integral formulation of the continuity equations [27]. These density mean field approaches have been recently put on a mathematically rigorous footing using results from probability theory [28]–[31].

Neuronal firing is inherently variable and the source of this variability has been subject to much study and debate [32]–[34]. Incorporating neuronal variability into theories is another level of complexity. Activity mean field models have been shown to exhibit complex dynamics with high variability when coupled with highly variable connectivity [35]–[38], but this is independent of single neuron dynamics. It is not clear in the context of the density mean field approach how to quantify the fluctuations arising from the interactions of discrete neurons in a finite-sized network, where the fluctuations are not suppressed by averaging over an infinite pool of neurons. *Ad hoc* attempts at quantifying finite-size effects include driving the system with external noise [13], introducing a self-consistent noise from neural firing [39], or assuming Poisson firing rates of the neurons within the population [17], [22], [40]. However, a systematic means of handling fluctuations due to the finite size of a population of neurons remain lacking.

Here, we present a systematic expansion around the density mean field behavior that quantifies the finite-size fluctuations and correlations of a population of neurons in terms of the interactions in the network. The expansion utilizes a kinetic theory approach adapted from plasma physics [41]–[46]. Because we are interested specifically in intrinsic fluctuations which arise across the population evolving via deterministic dynamics, we do not include any external “noise” or internal stochasticity. The network variability is thus entirely due to the fact that many possible neuron initial conditions and parameters are consistent for a given network, which implies that a given network is selected from an ensemble of networks. One should think of this ensemble as the ensemble of networks consistent with an initial experimental setup, or of those networks which are consistent with the experimentally accessible quantities in the network. In particular, we show that fluctuations and correlations and their effect on population behavior can be quantified in a fully deterministic dynamical system by considering the ensemble of system histories given a distribution of initial conditions and network parameters. In the finite size case, the density 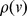 will not represent the fraction of neurons in the network with potential in the interval 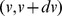 (as it is in the infinite neuron case), but will represent the fraction of networks in the ensemble for which there is a neuron within the interval 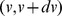. In the cases we consider, there is a “typical” system in the large neuron limit, so that the two are nearly identical. To a given order in the network size 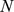, one can derive a moment hierarchy of differential-integral equations for the statistical moments of the density 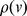. The calculations are facilitated by transforming the moment hierarchy into a functional or path integral expression of the moment generating functional from which a perturbative expansion can be derived. We show this for two synaptically coupled neural networks in the Results and provide some guidance on generalization to other models in the Discussion.

Our approach is thus in the spirit of Gibbs' view of statistical mechanics [47]. Like Gibbs, we do not rely on ergodicity or make any claims about time averages of the dynamics. The systems we study do not obey detailed balance and thus there will not be a necessary correspondence between time averaging and the ensemble averages we study. Nonetheless, we obtain useful results for characterizing the fluctuations and correlations in a network. We consider a specific example with global coupling where these correlations will have well-defined expansions in terms of the inverse systems size 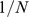 and we refer to them as “finite-size” effects. However, we wish to stress that our approach is not restricted to a finite-size expansion in 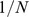 per se. Our main result is to provide a systematic framework to “average” over unknown or unessential degrees of freedom.

## Results

### The density description of neural networks

We present a formalism to analyze finite-size effects in a network of 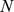 synaptically coupled spiking neurons. Under fairly generic conditions, such a system can be reduced to a set of phase variables with a set of ancillary variables (such as those representing synaptic input) [48]–[51]. We consider the phase dynamics of a set of 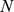 phase neurons obeying
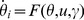
(1)

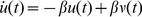
(2)

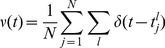
(3)where each neuron has a phase 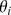 that is indexed by 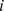, 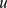 is a global synaptic drive, 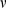 is the population firing rate of the network and 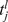 is the 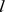
th firing time of neuron 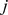 and a neuron fires when its phase crosses 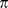. The frequency function 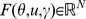 depends on all the phases 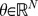 and a set of 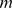 parameters 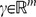, that can be distinct for each neuron 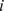. The neuron can be in an oscillatory or excitable regime.

We will develop our theory for a general frequency function 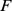 and apply it to the specific cases of a simple phase oscillator where 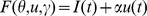 [10] and the theta model where 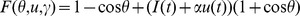, where 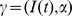, 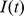 is an external input and 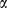 is a parameter that can be neuron dependent. The theta model is the normal form of a Type I neuron near the bifurcation to firing and is equivalent to a quadratic integrate-and-fire neuron [52]. For some neural networks, a phase reduction of this sort results in a phase coupled model, such as the Kuramoto model (e.g. Hansel and Golomb [53]), which we have previously analyzed [41], [42]. In the present paper, we consider all-to-all or global coupling through a synaptic drive variable 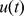. However, our basic approach is not restricted to global coupling.

Our goal is to derive the fluctuation and correlation effects beyond mean field theory for the system. For global coupling, these effects arise from the finite number of neurons 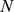 in the network. We calculate the effects of finite 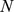 on the dynamics of the system as a perturbation expansion in 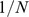 around the mean field limit of 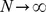. In particular, we will compute the fluctuations and correlations of the synaptic drive 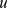 and network firing rate 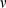, defined as the variability over instances of the network given initial conditions as well as neuron and network parameters. We will do this through a probability density functional description of the neuron firing histories. Before we introduce our density functional approach, we describe the Klimontovich description of many-body systems. This description allows us to introduce the fundamental degrees of freedom in a straightforward manner without recourse to the statistical field theory formalism used in the density functional approach. While we focus on finite size effects in this paper, our method could also be used to generate perturbation expansions in other parameters.

#### Klimontovich description

We adapt the methods of the kinetic theory as applied to gas and plasma dynamics to create a probabilistic description of the network dynamics [45], [46]. The approach will allow us to calculate the corrections to mean field theory due to correlations in the firing times of neurons. In particular, we employ a Klimontovich description, which considers the probability density of the phases of a population of neurons (i.e. the density of the *empirical measure*)
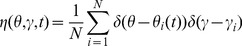
(4)where 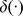 is the Dirac delta functional, and 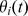 and 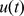 are the solutions to system (1)–(3). The neuron density gives a count of the number of neurons with phase 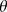 and parameters 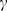 at time 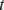. We have included the parameter vector 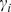 in the neuron density. Hence, neurons are characterized by their phase and parameter values. For systems that obey exchange symmetry or exchangeability (i.e. the system remains unchanged statistically after a relabeling of the neurons), the neuron density in (4) gives a complete description of the system. In systems without exchangeability, the neuron density will still capture the complete dynamics of the system if it includes labels for the information attached to individual neurons. Using the fact that the Dirac delta functional in (3) can be expressed as 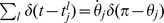 the population firing rate can be rewritten as
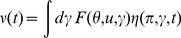
(5)The neuron density formally obeys the conservation equation
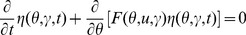
(6)which is known as the Klimontovich equation in kinetic theory and is only valid in the weak or distributional sense since 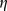 is not differentiable. The Klimontovich equation, the equation for the synaptic drive (2), and the firing rate expressed in terms of the neuron density (5), fully define the system. For the systems defined above, we expect that in the limit of a large number of neurons the ensemble of networks will converge to a “typical” network. In the infinite neuron limit, this will give the density equations of mean field theory, whereas for finite but large 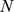, there will be some variation in systems around the mean field solution. For this reason, we consider taking expectations of the Klimontovich equation (6) over initial conditions and neuron parameters, which produce smooth moment functions for the density. Because the interacting dynamics have a non-trivial effect on the distribution functions, computing this average is not always simple. In the next section, we will formally derive an expression for the measure or density functional 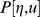 over which these averages are taken.

Denoting averages over initial conditions and neuron parameters (i.e. those over 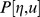) by 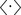, the average of (6) yields the equation
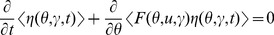
(7)where 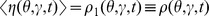 is the first moment of 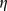 and called the one-neuron distribution function, which will depend on higher order moments since 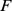 is a function of 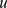 and hence 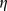. Equations for the higher order moments can be constructed from (6) by multiplying by factors of 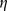. However, each moment will depend on yet higher moments, resulting in a system of coupled moment equations called the BBGKY hierarchy. Solving the entire BBGKY hierarchy is equivalent to solving the original system and thus provides no computational advantage. However, perturbative solutions in a small parameter such as 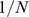 can be obtained by truncating the hierarchy and solving the truncated system. This has been the traditional approach in kinetic theory but is generally difficult to do. In the next section, we present a computational formalism where moments for the firing rate and synaptic drive are computed directly from a probability density functional of the neuron density.

Mean field theory is obtained by neglecting all correlations and higher order cumulants. Thus, setting 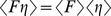 gives the self-consistent mean field system
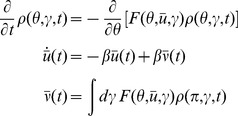
(8)Higher order moments (distribution functions) 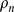 are likewise defined. The second moment (2-neuron distribution function), 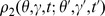, is the fraction of networks in the ensemble for which there is a neuron of type 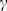 at 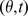 and another of type 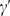 at 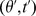. It is given by
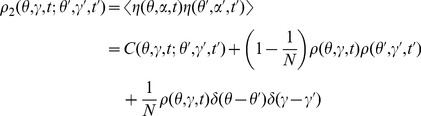
(9)We have implicitly defined the function 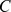 using the fact that if the neurons are prepared identically and independently, then 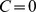. We call 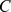 the *connected* contribution and the product of 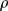
's the *disconnected* contribution. These labels are equivalent to whether the contribution can be factored into products of lower moments. The two-neuron density function has connected, disconnected and finite-size (those with factors of 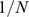) contributions. The finite-size contributions arise from the deviations in the ensemble average due to finite sample size. There are two types of finite size correction. There is a “sampling” correction because of the “diagonal” contribution where the indices 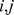 from the two factors of the neuron density 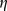 (4) coincide. Since 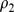 represents the joint probability density function of two neurons drawn from the population, there is a finite-size correction due to the fact that once a neuron has been drawn from the population, that neuron's phase 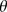 is fixed and the probability density for that neuron is a point mass at that phase. Thus, the sampling finite size term consist of removing 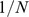
th of the joint probability mass from 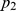 and adding it back as the one-neuron density multiplied by the Dirac delta functional. In the infinite 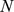 case, the probability of drawing a strictly identical neuron twice is zero.

The second type of finite size effect is due to the coupling and is contained in 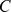 (it will be proportional to 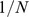). For uncoupled neurons, if the neurons are not prepared such that 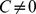, then no such correlations will be generated by the dynamics. Note that integration of 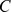 over 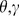 (or 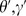) gives 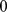. One can derive similar expressions for the higher moments, i.e. for the 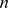-neuron densities. There will be connected terms which cannot be factored into products of lower moments, there will be disconnected terms which can be so factored, and there will be finite size corrections given by the combinatorics of drawing 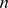 neurons from a population of size 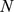.

### Density functional description

We have shown that one tractable approach for incorporating fluctuations and correlations is to truncate the BBGKY hierarchy. However, solving such truncated systems for any model of reasonable complexity quickly becomes unwieldy. For this reason, we adapt the density functional formalism developed for statistical field theory to obtain a formal expression for the probability density functional of the neuron density and synaptic drive 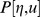. The fundamental degrees of freedom in this approach reflect the moments of 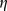, albeit in a more compact and manageable form. The measure 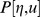 is a distribution over the possible network realizations. The “variance” of this distribution (represented by the two-neuron distribution function) provides an indication of the extent to which different realizations of the network will differ from each other. For the systems we consider, the estimates of the 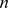-neuron distribution functions behave as a power of 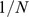. This has the side benefit of demonstrating that there is a limit in which the ensemble converges to a “typical” system described by the 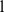-neuron distribution function, 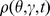, i.e. the mean field theory. For the same reason, at large 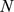, we can use the 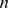-neuron distribution functions as estimates of the fluctuations in the density for a *single* system. Because these fluctuations vanish in the limit of large 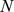, we term them “finite-size” effects. In the examples below, we concentrate on computing the 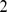-neuron distribution function to lowest order in 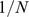, which gives estimates of fluctuations of the network coupling variables and the firing rate.

In this section we present only final results, the complete derivation and description of the computational method can be found in the Methods. The essential element of the field theoretic method is that the density functional be expressed in the form 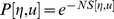, where 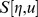 is called the *action*. Given this density functional, moments can be obtained by integrating over this density. For example, the second moment of 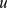 is given by a functional or “path” integral
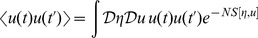
where the measure in the integral is over *functions* of 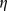 and 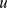 in some appropriate functional space. A generating functional for all the moments or cumulants can be similarly defined (see Methods). The strategy of field theory is to exploit the fact that Gaussian integrals have closed form expressions in an arbitrary (including infinite) number of dimensions. Hence, the path integrals can be performed using Laplace's method or the method of steepest descents to obtain an asymptotic series expression for the integrals in terms of a small parameter, which in this case will be 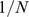.

In general, the action 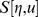 is not expressible in simple form. This is overcome by augmenting the system with an auxiliary set of imaginary *response functions* 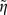 and 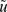 and defining an expanded action 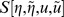. The action can then be Taylor expanded around a critical or saddle point where 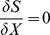 (where 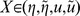), which produces an expansion of moments of a “Gaussian” distribution, in this case arising from the terms bilinear in the auxiliary variables 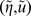 and the configuration variables 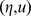. A perturbation expansion can then be constructed by exploiting the fact that complex Gaussian integrals of the form
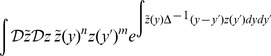
(for some variables 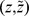) have closed form expressions in terms of linear response functions or propagators 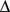 and are nonzero only if 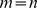. This path integral identity can be used to formulate an explicit set of rules to obtain expressions for each term of the perturbation expansion. The computation is simplified by encapsulating the rules for constructing the terms in the expansion into diagrams (i.e. Feynman diagrams, see Methods).

The variables in the action can be compared to those in a stochastic differential equation. The original variables (without a tilde, e.g. 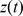) denote the configuration variables, while the auxiliary variables (with a tilde, e.g. 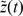), denote stochastic or noise forcing terms although in our case the noise is imposed by the uncertainty in the initial conditions and heterogeneity in a fully deterministic network. Finally, the method does not compute the action directly in terms of the neuron density 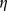 but rather transforms it to a new set of neuron density variables 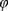 and 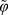 through the transformation 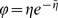 and 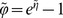. This transformation renders the action to be more amenable to analysis in a way that is similar in spirit to how the Cole-Hopf transformation reduces the nonlinear Burger's equation into the linear heat equation [54]. Specifically it removes the Poisson-like counting noise from the definitions of the moments. As an example, whereas
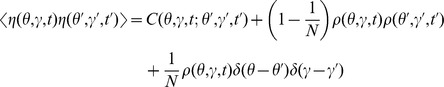
the transformed variables have
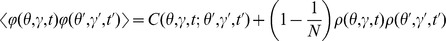
As discussed in Methods, the population level coupling implies that the desired quantities will have an expansion in powers of 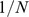. We describe basic results of this approach on two particular example networks: the phase model and the quadratic integrate-and-fire model. For each model, we describe mean field theory, the linear response of the population, and all the correlation functions involving the population and the synaptic drive. Each quantity is calculated to lowest non-trivial order.

#### Phase model

We first apply the formalism on the simple phase model defined by
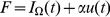
(10)where 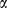 is the magnitude of the coupling of a given neuron to the global activity 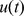 and 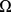 indexes the input. (In analytical terms, 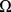 is an element of the sigma algebra representing the realizations of the inputs 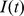, for example an instance of Brownian motion input).

The action for the phase model as derived in the Methods has the form
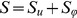
(11)where 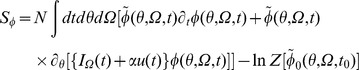
represents the contribution of the transformed neuron density to the action and 
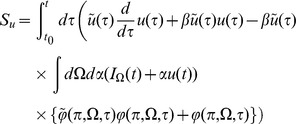
represents the global synaptic drive. The action (11) contains all the information about the statistics of the network. Given the action, mean field theory and a perturbative expansion around mean field theory can be derived using standard methods developed in field theory.

The mean field equations, which are given by a critical point of the action, are given by (8), which for parameters 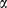 and 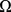 are rewritten as
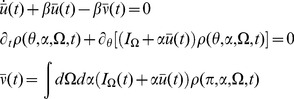
(12)For the phase model, we can solve (12) directly for 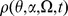 to obtain
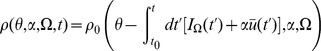
where 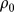 is the initial distribution. In this case, the functional form given above is also the general (non-mean field) solution, upon replacing 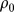 with 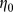. Recall that 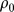 is the population distribution averaged over the ensemble of prepared networks. If the neurons are distributed uniformly in phase, then 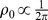. In this case, the global activity does not affect the phase distribution. On the other hand, if the neurons are always prepared at the same phase, then 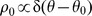, where 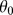 is the prepared phase. In this case the neurons will remain in phase.

Solving for 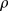 allows us to write a closed integro-differential equation for the synaptic drive
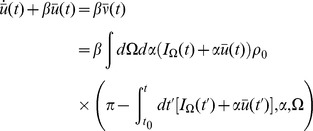
Note that as long as 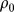 is known, this mean field equation reduces the system from a partial differential equation to a two dimensional ODE, namely:
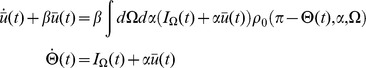
The population behavior is reduced to the synaptic drive dynamics along with the dynamics of a fictitious oscillator 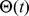. This is the result of the fact that the only important dynamical quantity is the overall phase shift of each neuron from its initial phase and that this quantity is the same for each neuron. Knowing the initial distribution of states is therefore enough to reduce the dimensionality of the system.

The steepest descent expansions to the path integrals will be expressed in terms of the propagators or linear response functions 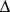, which appear as the inverses of the integral kernels of the bilinear terms in the actions. The linear response can be derived to order 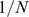 by linearizing about the solutions of the mean field equation. Because there are two fields in the action (synaptic drive and density), there are four separate propagators:
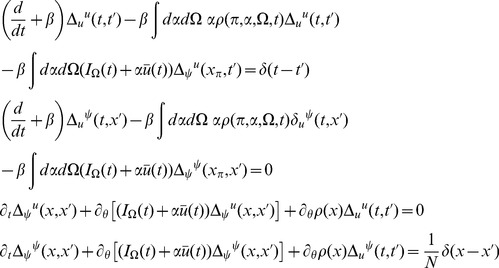
(13)where 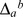 describes the response in the quantity 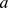 to a perturbation in the quantity 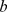, 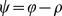 denotes perturbations around the mean field solution, 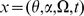 and 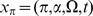. The equations for 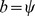 reflect a perturbation that consists of adding a single neuron to the population with the specified initial condition and parameters.

If we assume a constant input 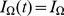 then in order to have a steady-state, the mean field must satisfy
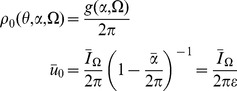
(14)for a fixed parameter probability density 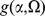, where 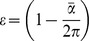, and 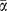 and 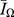 are the means of 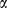 and 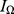 under the distribution 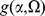. The linear response around this solution is
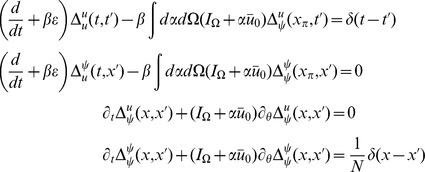
which we can immediately solve in closed form to obtain
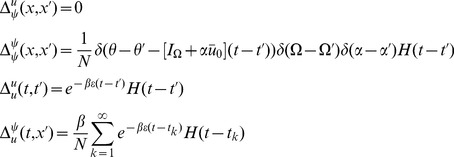
(15)where 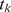 are the firing times of the fictitious oscillator 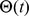 with initial condition 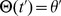, and is determined by 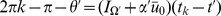. 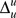 is the expected form of the linear response upon perturbing the synaptic drive 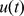, i.e. exponential decay. The response of 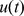 to the population density 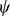, 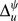, is a series of exponential pulses at the firing times of the additional neuron, which is what we would expect if we added a single neuron at a given phase. The other propagators govern the response of the population. Since the distribution is uniform and the firing rate does not depend upon phase, perturbing the synaptic drive only makes the entire population fire faster, but does not change the relative phase, thus 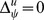. On the other hand, adding a single neuron adjusts the population density by a single delta function at the location of the new neuron, hence the form of 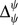. The fact that single oscillator perturbations are not damped away by the linear response is an indication that the stationary state is marginally stable. We expect that finite size effects at the next order will stabilize these marginal modes assuming there is some degree of heterogeneity similar to what happens in the Kuramoto model [41], [42].

As described in Methods, the expansion of any 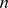-neuron correlation function in powers of 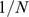 can be computed from the linear response and the “vertices” derived from the action 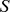. Here we give the lowest order contribution to the 2-neuron correlation functions. In addition, this will give us the firing rate fluctuations. For the fluctuations in the synaptic drive 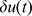 about an arbitrary mean field state 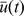, the diagrams at tree level (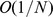) give
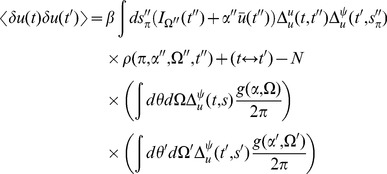
(16)where 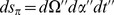. Inserting the expressions for the linear response in the stationary state (15) we obtain :
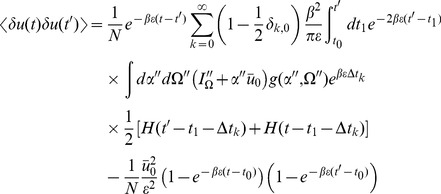
(17)for 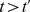, where the 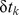 are determined by 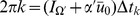 and 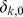 is the Kronecker delta. The reason for the Kronecker delta term is to account for the limiting process which defines the interaction vertex. Essentially only half the neurons within the vicinity of firing will contribute to the first cycle of firing (about half are above threshold, half under). On subsequent cycles, all neurons will contribute. This issue arises here because of an ambiguity of the continuum representation we are using. The vertex only measures those neurons which have passed threshold, whereas the linear response from (15) considers the limiting behavior of neurons initially configured in the neighborhood of some phase 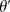 (consider the last equation in (15)). If the distribution 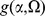 is smooth, it is more convenient to compute the term 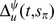 convolved with the function 
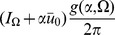
.

Performing the time integration gives
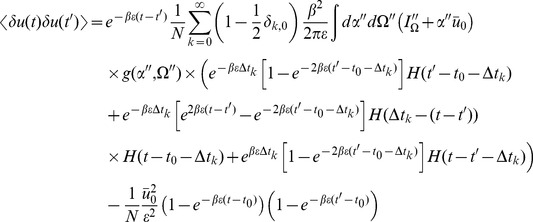
(18)The equal time correlation function has a simpler form:
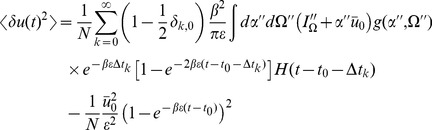
(19)This correlation function quantifies the fluctuations in the global coupling variable 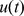 as a function of time. Recall that we defined an initial state in which each neuron is statistically independent in phase and parameters. The time 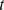 is the interval elapsed since the network was in that initial state. 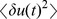 is a measure of the expected variance of the synaptic drive from the mean 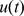 at time 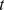. As mentioned above, due to the fact that higher moments of 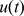 will be suppressed by higher powers of 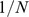, this is also an estimate of the variance of the global coupling as a function of time 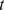 from a *known mean field configuration*. Because the linear response has a spectrum which includes the spectrum of the single neuron activity, we expect behavior characteristic of the time scales of single neuron dynamics to appear.

We now turn to the correlations in the density variable 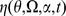. As discussed in the Methods section, these are given by (let 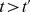 and 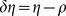)
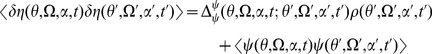
(20)The first term is given by expressions derived above. The second term is of the same form as the correlation of the synaptic drive variable.
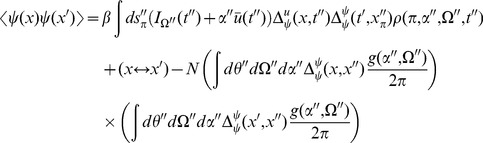
(21)The above is the general expression. For the fluctuations about steady state, 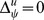 from (15), giving the simple relation
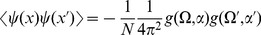
(22)which is just the negative of the product of the mean field steady state solutions at each argument 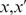 times a factor of 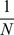. This term is due to the factor 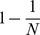 from the sampling correction in the two-neuron distribution function (see equation (9) and below).

The 2-neuron distribution function is given by
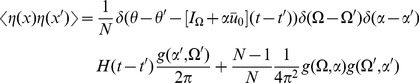
(23)At equal times (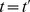) we have
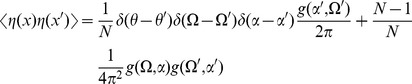
(24)which shows that (22) is the correction term for the normalization of the two-neuron distribution function. So for the case of the simple phase model, the fluctuations in the density about steady state are given by the sampling fluctuations from the steady state distribution. Note that for large 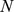 this means that the variance of the number of neurons at firing (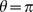) is equal to the mean times a factor of 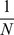, which is equivalent to the Poisson counting assumption of Brunel-Hakim [17]. As we will show in the next section, this will not hold in general. Note the form of the linear response (15) for the term 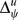. The fact that the linear response 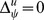, eliminated the first term in (21), which is the contribution to the fluctuations from the coupling. Comparing to the general form of the linear response (13), we see that the equation for 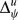 has a source term proportional 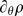. Because the phase model has a uniform steady state, this source term is zero. For a model with a non-uniform steady state (such as the quadratic integrate-and-fire model, which we examine in the next section) this will not be the case, and there will be further corrections to the fluctuations in 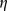. It occurs in the phase model because perturbations in the synaptic drive do not perturb the density in steady state. Thus the only fluctuations of the density in steady state are from the sampling fluctuations.

The correlation function between the global coupling and the density is given by (with 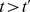).
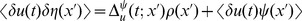
(25)Again, the first term is composed of factors derived above. The remaining unique term is given by
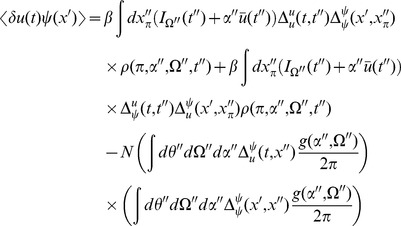
(26)In steady state, this term is
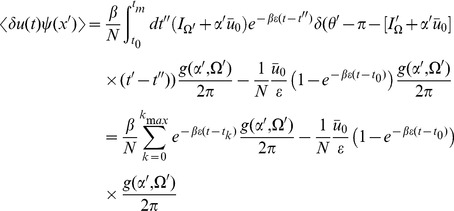
(27)where 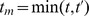, the 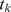 are defined such that 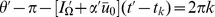, and 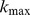 is the largest 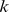 such that 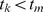.

The firing rate of the population is given by
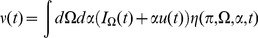
(28)The mean field solution for this is
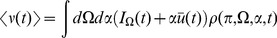
(29)and in steady state we have
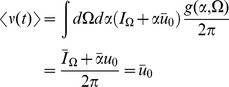
(30)The second moment of the firing rate is given by
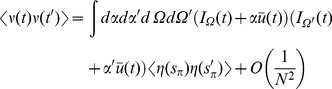
(31)Using our expression for the variance of 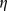 in steady state, we have
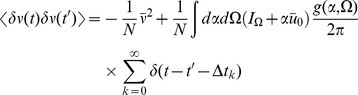
(32)where 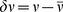 and the 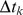 are such that 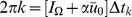. At equal time we have the simple form
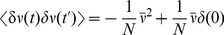
(33)which is equivalent to the Poisson finite size ansatz. The delta function evaluated at zero is a singularity which arises upon attempting to isolate a counting process at a single point on the real line. This can be regularized by considering an estimate of this quantity in a time interval 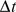. The variance in the counts will vary as
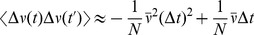
where 
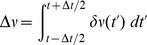
. This indicates that the population firing rate will appear as that from a population of independent Poisson neurons even though the individual neurons are regular. For intuition as to why this is the case, consider dividing up the interval 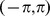 into bins of equal size and distributing 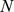 neurons into these bins. This is the initial state of the network when initialized in steady state. The distribution of the neuron counts in each bin will follow a hypergeometric distribution. In the limit of small bin size and large 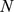, the number of neurons in each bin will approximate a Poisson distribution. The factor of 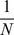 arises from normalizing the coupling by 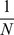. Recall that the absence of any other correction is an artifact of the uniformity of the steady state of the phase model. This will not be the case for the quadratic integrate-and-fire model.

**Figure 1:**
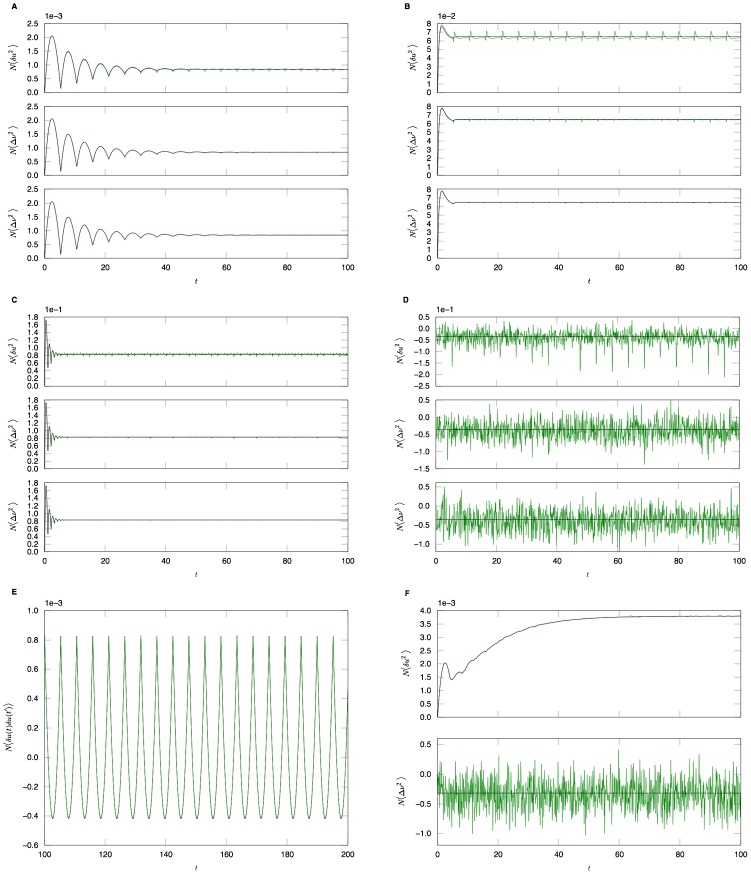
Phase model. **A**. Numerical computations (green line) and analytical predictions (black line) for 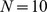 (top), 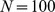 (middle), 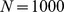 (bottom) of 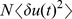 for 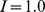, 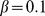, 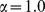. **B**. Numerical computations (green line) and analytical predictions (black line) for 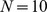 (top), 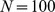 (middle), 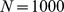 (bottom) of 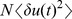 for 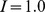, 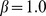, 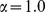. **C**. Numerical computations (green line) and analytical predictions (black line) for 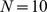 (top), 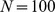 (middle), 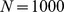 (bottom) of 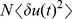 for 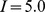, 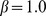, 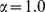. **D**. Numerical computations (green line) and analytical predictions (black line) for 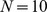 (top), 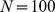 (middle), 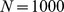 (bottom) of 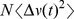 for 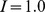, 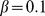, 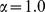, where the “Poisson” contribution has been subtracted. **E**. Two-time correlator 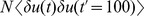 for 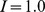, 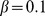, 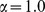, and 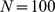. **F**. Equal time correlators in a heterogeneous network; 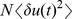 and 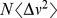 for 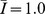, 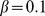, 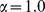 and 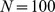. 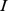 is taken from the interval 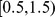 for each neuron. Ensemble averages for all simulations are taken over 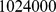 samples.

Figure 1 shows comparisons between our analytical predictions and numerical simulations. In (a) through (d), the network the parameters 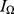 and 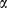 are constant and homogeneous (i.e. 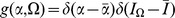). Figure 1 (a)–(c) shows examples of the variance of the synaptic drive as a function of time. As seen in the figures, the correlation function has contributions that appear at the firing times of the fictitious oscillator 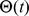 (Recall that 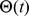 is a function which parameterizes the linear response). Each such “firing event” produces a new positive transient response in the correlation function. As 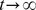, each firing event produces ever smaller perturbations as the correlation approaches steady state. Note also in those figures that the analytic computation at order 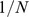 becomes better as 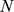 grows larger, and that the overall magnitude scales as 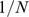. Deviations are observable for small 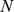, particularly for the case 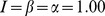. Note also the firing rate of the fictitious oscillator increases as the population input increases. Comparison of numerical and analytic results for 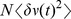 is shown in Figure 1 (d). We measured this quantity by binning the firing counts in a time window 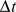 and have also subtracted the “Poisson” contribution. The analytic result is the first term from equation (33). Figure 1 (e) shows the two-time correlation function 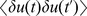, where we have fixed 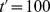. As expected by our prediction in equation (18), the oscillations are much more pronounced. Figure 1 (f) shows the effects of heterogeneity on the synaptic drive. The drive distribution was chosen to be uniform, with inputs to each neuron chosen from the interval 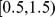. The oscillations in the synaptic drive are damped by the heterogeneity and there is an effective increase in the mean drive fluctuations as expected from the theory. In this case the heterogeneity clearly dominates as a contribution to the fluctuations, as can be seen by comparing figures 1 (a) and 1 (f), which differ by close to a factor of four in steady state.

### The quadratic integrate-and-fire model

The second model we analyze is the quadratic integrate-and-fire model, whose single neuron dynamics are given by
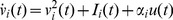
(34)This model exhibits a finite-time blow-up that is considered to be “firing” at which point the neuron's membrane potential 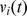 is reset to 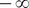. We couple the neurons in the same manner as in the phase model with the synaptic drive 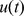. Ermentrout and Kopell mapped this model to an oscillator using the transformation 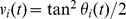 [55] to obtain
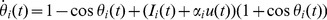
(35)This form of the model is often called the theta model [55]. Hence, the function 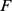 is given by:
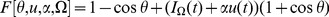
(36)A convenient feature of this model is that neurons cross the firing phase 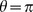 at a constant rate 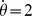.

Defining the neuron density in the same way as before
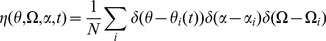
(37)the continuity equation is
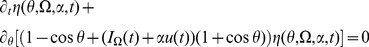
(38)The action, constructed according to the procedure outlined in the Methods section, is
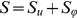
(39)where the population part of the action is
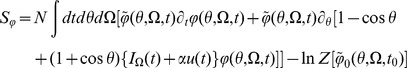
and the part representing the synaptic drive is
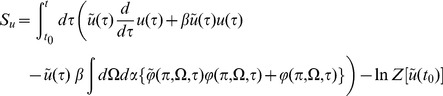
(40)Mean field theory is given by
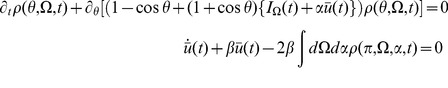
(41)Note that because the firing rate is constant at 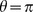, the input to the synaptic drive is only dependent upon 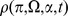 and not directly on the synaptic drive itself.

It is useful to examine the steady state of this model in some detail. For a constant drive 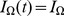, the steady state obeys
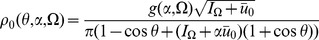
(42)For 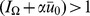, this solution is a unimodal distribution peaked at 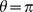 whose width narrows in proportion to the size of the input. Conversely, for 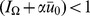, the peak is at 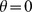. The higher the input, the more likely it is that any given neuron will be found near the firing phase, 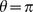. The synaptic drive variable must satisfy a consistency condition:
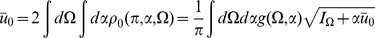
(43)This equation can be viewed as the steady state solution to a Wilson-Cowan type rate equation. The firing rate for the quadratic integrate-and-fire model is given, in the mean field approximation, by
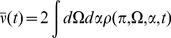
(44)In steady-state we have
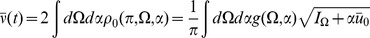
(45)so that we can identify 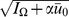 as the “gain” function for the neurons of type 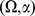.

The linear response for the coupled theta model is given by the equations:
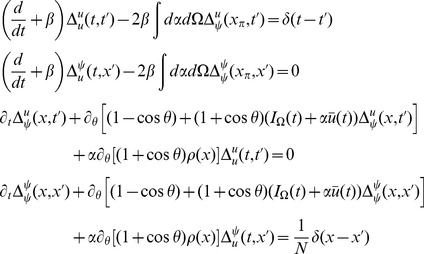
where again 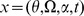 and 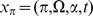.

Consider the steady state and transform the angle variable for each 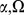 with
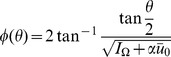
(46)Then we have
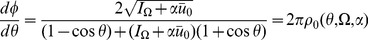
(47)This change of variables makes the steady state uniform in 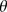 for each 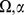. The equations for the linear response in steady state in terms of 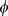 are
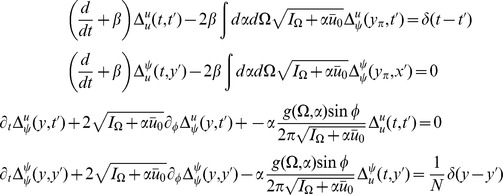
where 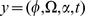 and 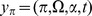 (note that 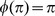).

The linear response for the theta model is most easily expressed in terms of the Laplace variable 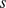 and is given by
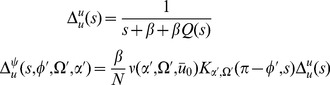
(48)where
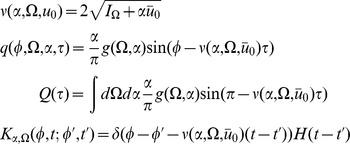


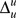
 is similar to the linear response of the synaptic drive in the phase model with the addition of the feedback response of the population through the filter 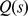. 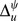 is the same as in the phase model with this transformation. It is a series of pulses with the pulse shape given by the linear response and the pulse times determined by the firing times of a fictitious oscillator driven at rate 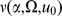.

We also have
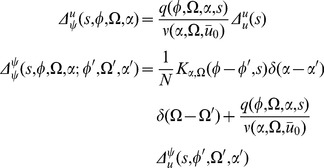
(49)These results produce the primary qualitative difference between the phase and the theta models. The first term in 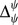 is analogous to the phase model calculation. It represents a perturbation of adding a single oscillator with initial coordinate 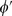 evolving at rate 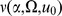. The second term and the non-zero value of 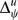 arise from the non-uniform distribution of the steady state, which arises from the functional dependence on 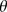 of the neural input function. This term produces deviations from the “Poisson” behavior of the firing rate fluctuations.

We can use these expressions to compute the tree level correlations with:
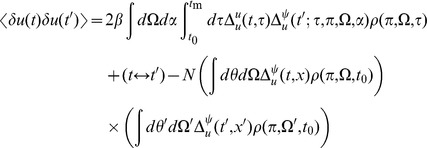
with 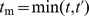. The other correlation functions are given by
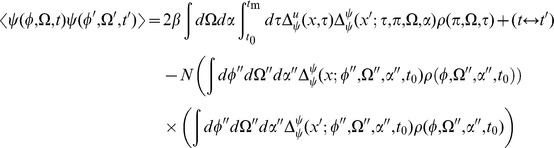
and
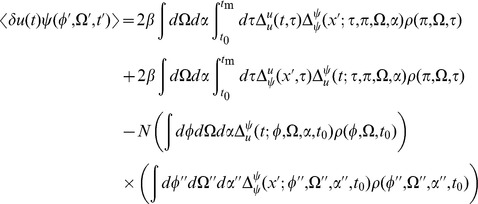
These are more difficult to put in closed form, other than in terms of the response function for the synaptic drive. Instead we show numerical results.

We can use the linear response formulas above to compute analytic formula for steady state. Changing coordinates and using the steady state mean field values we have
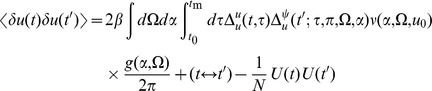
where the Laplace transform of 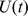 is given by
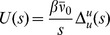
and
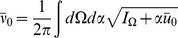
is the firing rate of the population in steady state. The correlations in the synaptic drive variable has the same basic form as that of the phase model. Because of the structure of 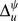 it will also have the same pulse behavior at an interval defined by a fictitious oscillator evolving according to the population activity. The primary difference is the replacement of the response function for the synaptic drive with the response for the theta coupling and the firing rate with the theta model firing rate.

The two-neuron density function, by contrast, is different by virtue of the non-uniform nature of the steady state. In this case, 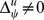 so there will be a contribution at first order in the perturbation expansion (i.e. tree level) to the density fluctuations. Similarly, there is an extra term for the correlation function 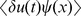. Each of these correlation functions is only computable in closed form in terms of the response functions, which we compute numerically.

**Figure 2:**
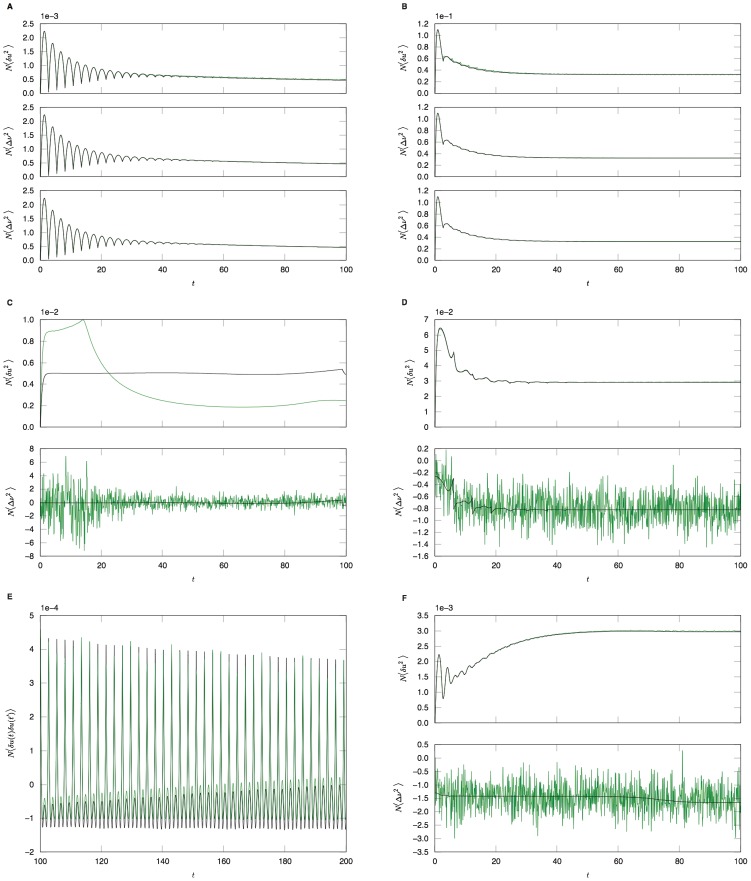
Quadratic integrate-and-fire model. **A**. Numerical computations (green line) and analytical predictions (black line) for 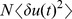 for 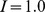, 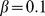, 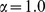 for 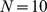 (top), 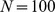 (middle), 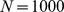 (bottom) neurons. **B**. Numerical computations (green line) and analytical predictions (black line) for 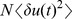 for 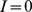, 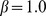, 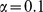 for 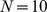 (top), 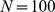 (middle), 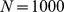 (bottom) neurons. **C**. Numerical computations (green line) and analytical predictions (black line) for 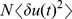 (top) and 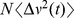 (bottom) for 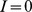, 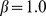, 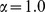, 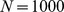. **D**. 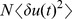 (top) and 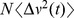 (bottom) for 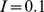, 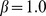, 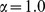, 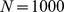, where the Poisson contribution has been subtracted. **E**. Two-time correlator 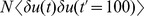 for 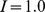, 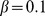, 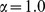, and 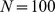. **F** Equal time correlators in a heterogeneous network; 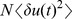 and 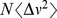 for 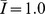, 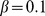, 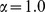 and 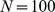. 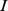 is taken from the interval 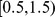 for each neuron. Ensemble average for all simulations are taken over 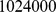 samples.

**Figure 3:**
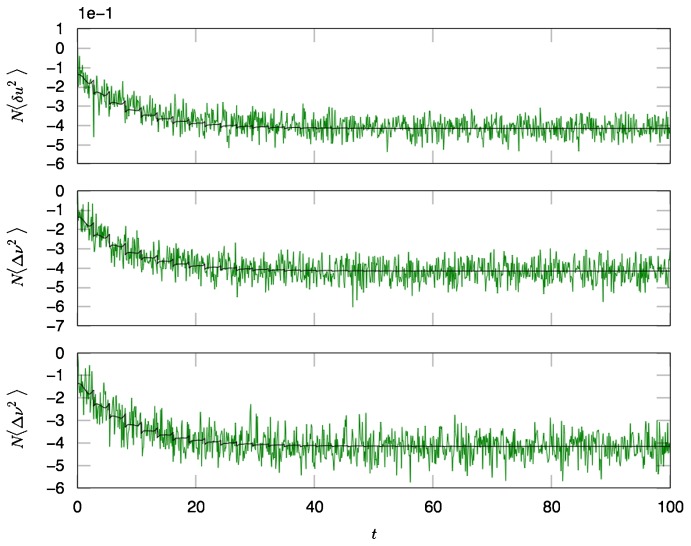
Numerical computations (green line) and analytical predictions (black line) of the firing rate fluctuations 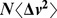 for the quadratic integrate-and-fire model for 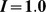, 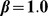, 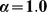 for 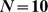 (top), 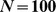 (middle), 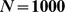 (bottom) neurons with Poisson contribution subtracted. Ensemble average is taken over 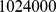 samples.

The firing rate fluctuations for the theta model are simpler than the phase model because the input for each neuron is the constant 2 at 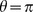. For the firing rate obeying
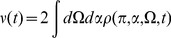
(50)the second moment of the firing rate is
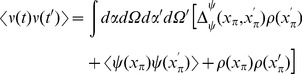
(51)for 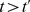. The equal time second moment is given by
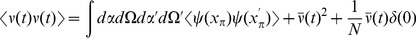
(52)where the 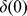 term has the same meaning as in the phase model. In the phase model case, the analogous expression to the first term on the right hand side was zero, and the population firing rate appeared to be the firing rate of the average of 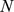 Poisson firing neurons. In the theta model case, however, there is a correction of order 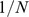. From (52), it is simple to show that the firing rate fluctuations in a bin of size 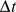 obey
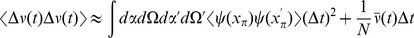
(53)Comparisons between analytic and numerical results for the quadratic integrate-and-fire model are given in Figure 2. In (a) through (e), the parameters 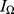 and 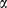 are constant and homogeneous. One can see the qualitative similarity between the phase and quadratic integrate-and-fire models in the behavior of the activity correlations, 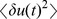. Both share the same pulsatile behavior driven by the fictitious oscillator, i.e. both show the spectral characteristics inherited from the single neuron dynamics. The density fluctuations, however, have an effect on the fluctuations in the firing rate. These effects can be seen in Figures 2 (c), (d). In addition to the nontrivial firing rate fluctuation dynamics, the quadratic integrate-and-fire model also shows near-critical behavior, owing to the phase transition between the “asynchronous state” and synchronous firing. For a population with no external drive, this transition occurs at 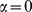. With 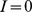, as in Figure 2 (c,) this represents a configuration in which the system is usually not firing, but with the occasional neuron moving across threshold. The reader is encouraged to draw an analogy with “avalanche” dynamics, in which the population will briefly fire in bursts and then go silent. While there is a small but fixed average firing rate, the fluctuations are large owing to this transient behavior. Even a small drive will regularize the system, as in Figure 2 (d). The finite size expansion is expected to break down near a phase transition, accordingly here it is expected to breakdown at the onset of synchrony. The breakdown of the expansion is evident in Figure 2 (c), where one can see enormous discrepancy between the analytic and numerical computations. Figure 2 (e) shows the two-time correlation function 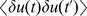 where 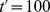. Figure 2 (f) shows the effects of heterogeneity on the synaptic drive, where the drive distribution was chosen to be uniform, with inputs to each neuron chosen from the interval 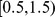. The oscillations in the synaptic drive are damped and there is an effective increase in the mean drive fluctuations as expected from the theory. Again the heterogeneity is the dominant contribution to the fluctuations, as can be seen by comparing figures 2 (a) and 2 (f), which differ by close to a factor of six in steady state. Figure 3 shows a comparison of the firing rate fluctuations. In contrast to the Phase Model, there is non-trivial temporal behavior owing to the phase dependence of the neuron dynamics.

## Discussion

We have constructed a system size expansion for the density formulation of spiking neural networks and computed the fluctuations and correlations of network variables to lowest order. In particular, we explicitly calculate two-neuron and higher order moments in the network. We have demonstrated our method in globally coupled networks with two different neuron types. We note that all the fluctuations and correlations are “finite-size” effects, i.e. they do not exist in mean field theory. There will also be finite-size effects on the mean firing rate and synaptic drive, which could also be calculated using our methods. However, in the systems we studied, the finite-size corrections to the mean field density in the steady state are necessarily zero by neuron conservation. The steady state is uniform and the fluctuation effects will not (for these models) break the symmetry.

The method is based on the Klimontovich equation, which is an exact formal continuity equation for the finite-size neuron density. Solutions to the Klimontovich equation only exist in the weak or distributional sense because the neuron density is a collection of Dirac delta functionals and is not differentiable. In the limit of infinite system size, it can be shown that under some conditions, the neuron density becomes a smooth function that obeys a strong continuity equation called the Vlasov equation [45], [46] that describes the mean field dynamics of the system. Previous work on large networks of coupled oscillators took the infinite system size limit immediately and started with the Vlasov equation [11], [15], [16]. If the oscillators are subjected to white noise, then the Vlasov equation becomes the McKean-Vlasov equation [12]–[14], [54], [56], which has sometimes been erroneously called a nonlinear Fokker-Planck equation. Recent work has put these density mean field methods onto a rigorous mathematical footing [28]–[31]. These authors prove that under reasonable assumptions, a network of stochastically coupled neurons under various conditions conditions will obey the McKean-Vlasov equation (Vlasov equation with diffusion) in the mean field limit. The network obeys the “propagation of chaos” property where neurons that are initially statistically independent will remain independent and the fluctuations are purely Gaussian. They also show that a self-consistent set of moment equations for the mean and variance when stochastically forced.

Our approach is based on the traditional Gibbs picture of statistical mechanics, to wit: the variability in the dynamics (in the absence of externally supplied noise or explicitly probabilistic dynamics) is a reflection of the distribution of “microscopic dynamics” which are consistent with the “macroscopic dynamics”, population level variables such as the global coupling, 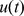. The fluctuations in the firing rate 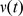 arise from the variability across neuron distributions which are approximately consistent with the mean field value. Those variables which converge to well defined values as 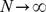 define the set of “macroscopic” variables. In the examples we have shown, the global coupling 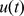 and the population density 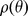 are considered macroscopic. In a more general network, such as one with heterogeneous coupling, the identification of macroscopic variables is likely to be a more complex issue. Put another way, in our simple cases there is a clear sense of the “typical” system for large 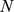 to which all initial conditions and parameters approach. There is no general requirement that “typical” systems exist.

The Gibbs picture is realized by taking the ensemble average of the Klimontovich equation, which leads to a moment hierarchy where lower ordered moments (or cumulants) of the neuron density depend on higher order moments. The moment hierarchy is an exact ensemble averaged description of the finite-size system. However, in general, solving the moment equations is as difficult if not more difficult than integrating the original system directly. For systems with a well defined large 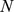 limit, the moments, such as the two-neuron correlation function, represent the finite size effects. Estimates for the moments can be obtained by truncating the moment hierarchy and solving a reduced system of equations, wherein 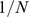 is a natural expansion parameter.

A truncated moment hierarchy is still unwieldy to solve. Our approach is to compute the moments directly by constructing a formal expression for the probability density functional of this distribution. This density functional is a “doubly” infinite dimensional object since its elements are infinite dimensional functions. Its formal construction hinges on the fact that it is proportional to a point mass (in an infinite dimensional functional space) located at a population density function that obeys the Klimontovich equation. Intuitively, this can be thought of as a Dirac delta functional with the Klimontovich operator as an argument. This expression is rendered computationally useful by noting that a Dirac delta functional in infinite dimensions has a Laplace transform representation where the integration is over a space of functions or fields and a set of imaginary response fields corresponding to the Laplace transform variables. The exponent of the integrand is called the action and fully specifies the distribution over the neuron density and synaptic drive.

Methods developed in quantum and statistical field theory are then employed to construct perturbative expansions for desired quantities such as moments. The expansions use the infinite dimensional analogue of the method of steepest descents. The action is expanded around a critical point at which the gradient is zero. The critical point condition yields mean field theory. The first order correction, or tree level, expands the action to quadratic order yielding an infinite dimensional Gaussian integral. The integral has a closed form expression in terms of the inverse of the Hessian matrix, which is analogous to the inverse of the covariance matrix of a finite dimensional normal distribution. Just as in a finite dimensional steepest descent expansion, the terms in the perturbative series will be in terms of the elements of the inverse of the Hessian matrix, which in our case correspond to the equations satisfied by the linear responses. Hence, the perturbative expansion of the time dependent moments of the coupled network will be in terms of the linear responses.

Previously, we applied this strategy to the Kuramoto model where oscillators are coupled directly through their phase differences. The corresponding action is a function of the population density together with the response field. The linear response satisfies the linear Vlasov equation. The tree level expression for the second moment of the population density, which captures the fluctuations due to finite-size effects, is identical to a solution of the truncated moment hierarchy known as the Lenard-Balescu solution in plasma physics [45]. Here, we consider a network of neurons coupled via synapses that are triggered whenever a given neuron fires. Hence, the field theory now involves the density and synaptic drive fields with their auxiliary fields. There are now four linear response functions, which makes the computations more complex.

Finite size effects were considered by Brunel and Hakim [17]. They assumed that the connections were sparse enough so that the arrival times of synaptic events at a given neuron would be uncorrelated. They then assumed that these inputs could be modeled by a Poisson process that was scaled by the number of inputs. We considered the opposite regime of a fully connected network. We find that for the phase model, the Poisson ansatz is essentially correct to order 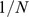. The theory of coupled diffusions in probability theory provides an explanation called “propagation of chaos” where the uncertainty in the initial conditions is propagated forward by the deterministic dynamics of the system [54], [56], [57].

Our approach generates a natural explanation for Poisson like firing rates in a population of neurons. Indeed, it is a natural consequence of the neurons firing in a stable asynchronous state. The number of neurons firing is the number of neurons out of 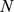 randomly chosen that fall into a small bin of size 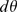 around the firing threshold. In the limit of large 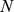, this should follow a Poisson distribution. For this reason, Poisson firing of the population is a natural assumption. However, as we have shown, if the neurons have some phase dependence in their voltage evolution, this will produce fluctuations in the firing rate beyond the simple sampling induced Poisson fluctuations.

The mean field theory for our system is comparable to a differential equation form of the spike response theory [27]. The use of phase oscillators allows for a continuity equation without a jump condition at the boundaries in a threshold crossing integrate-and-fire neuron. It may be possible to perform a similar finite size expansion within the spike response theory by incorporating the boundary conditions. The mean field equations have Wilson-Cowan rate equation form in that all the inputs to population activity enters through the firing rate function. This arises because of our choice of the global synaptic drive dynamics where the synaptic inputs of the population are first summed and then “filtered”. If we had instead chosen synaptic dynamics such that the synaptic inputs are first filtered and then summed, we would arrive at the “Amari” formulation of the mean field equations in which the external inputs to the activity equation lie outside of the rate function.

We considered the example of an all-to-all network. In this case, the 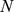-neuron joint distribution for the network obeys exchangeability, which means that the marginalization of the distribution over any set of 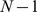 neurons yields the same distribution. For such a system, the neuron density function is a complete description of the network. However, we can always write down a neuron density function for any network even if it does not posses an exchange symmetry. For such a situation, the density function still captures useful global dynamics of the network. In the case of heterogeneous neuron parameters, as we considered here, the network is exchangeable in the infinite 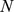 mean field limit and close to exchangeable for large but finite 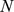. Hence, our formalism is directly applicable in this case. Such networks are said to be “self-averaging” in that the large network can be divided into sub networks, whose average behavior mirrors the full network. However, the situation with heterogeneous connection weights is more complicated. In such a system, it is not certain that the network is self-averaging in the infinite 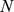 limit. If so then the mean field equations are not a useful description of the system. An analogy can be drawn to spin glasses, where depending on parameters, the system may or may not be self-averaging. The conditions under which a heterogeneous network of spiking neurons is self-averaging is a question that we wish to pursue in the future.

However, even in the case of a heterogeneous network without self-averaging, we can still apply our formalism if we consider the network to be comprised of local populations which exhibit exchangeability [28]–[31], [43]. In this case, each local population would be represented by its own neuron density, which are then coupled to other neuron densities. Each local density would obey its own Klimontovich equation and corresponding moment hierarchy. If the local populations are sufficiently large then the hierarchies can be truncated in a finite-size expansion as shown here. However, even if the local populations are not large or even consisting of a single neuron, our formalism could still be applied. A moment hierarchy or density functional for the entire system could still be constructed. Although a perturbation expansion cannot be constructed using the inverse system size as a small parameter, an expansion could still be constructed using some other small parameter such as the inverse of a slow synaptic time constant or the inverse of the number of connections. The mean field limit would consist of a network of coupled local activity fields. This could then be generalized to a network of coupled moment equations such as the activity and correlations. We had previously derived generalized activity equations for an abstract spike count model [43].

There is always a tension in computational neuroscience between detailed realistic models versus simpler reduced models. The main purpose of this work is to build quantitative tools to bridge the gap between the two approaches. We have developed a principled method of coarse-graining a neural system that is relatable to experimentally accessible quantities. Even with the exponential increase in available data and computational power, detailed realistic modeling will still have limitations. For one, a large scale simulation of the brain may not necessarily be easier to understand than the brain itself. An exhaustive exploration of parameter space will be intractable even if Moore's law holds up for centuries. Thus, there will always be a role for theoretical analysis of simple models. However, one of the criticisms of reduced models is that they are *ad hoc* and cannot be easily linked to the underlying physiology. Hence, there is a need for methods to derive reduced models directly from detailed models. Additionally, one would also like to derive reduced models that can incorporate single neuron effects such as synchronization and correlated firing, which are lost in classical mean field models. This motivated our desire to derive generalized activity equations that include such discrete neuron effects. Applications for generalized activity equations include studying the effects of correlation-based learning rules as seen in spike-timing dependent plasticity, understanding the role of oscillations in motor and sensory processing, and probing the neurophysiological basis of cognitive disorders by analyzing how perturbations to neural parameters affect cortical circuit function.

## Methods

### Action and generating functionals

The population statistics of the network is encoded in a hierarchy of moment functions of the population density, 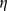 and the synaptic drive 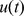. We now show that these moments can be systematically encoded into a generating functional specified by an action, from which each can be calculated via perturbation theory. The system is fully specified by Equations (2), (5), and (6), which we rewrite as
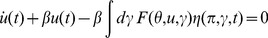
(2′)

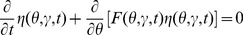
(6)where we have substituted (5) into (2) and the equations are subject to appropriate initial and boundary conditions.

We wish to derive a probability density functional 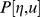 for the dynamical variables 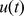 and 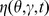, from which we can derive all statistical measures for the network dynamics. We can factorize the density functional into 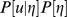 and compute the probabilities separately. The density functionals are usually represented in terms of an action, which for the networks we consider are given by (11) and (39).

The derivation is applicable to any dynamical system, so we derive it for a generic variable 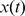 that is governed by the differential equation
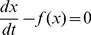
(54)with an initial probability density for 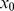, 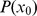. The dynamical system is fully deterministic and the density functional will describe the ensemble of many such systems starting from different initial conditions. Given the probability density at time 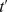, the probability density at a later time 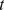 can be written as
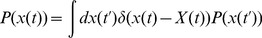
(55)where 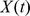 is the solution of the dynamical system (54) with fixed initial condition 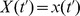.

The generating function for the moments of 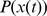 is given by the Laplace transform of 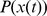:
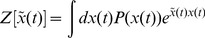
(56)where the variable 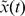 is called the “response field”. The moments are obtained from the generating function by taking derivatives with respect to 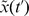 and setting 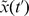 to zero. The natural log of the generating function is called the cumulant or connected generating function. Derivatives of 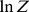 generate cumulants, i.e. those contributions to the moments which cannot be factored into products of smaller moments. The nonlinear terms in 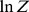 therefore represent the “noise” or correlations in the distribution being represented. For example, the connected generating function for a Gaussian with mean 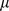 and variance 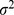 is 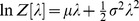 and for a Poisson distribution with mean 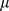 it is 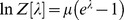.

Inserting (55) into (56) yields
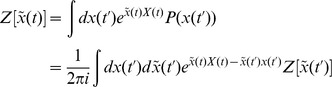
where we have used the inverse Laplace transform for 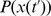. Setting 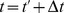 and taking 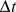 to be much smaller than any time scale in (54) allows us to write the solution as an Euler step
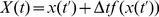
(57)which leads to
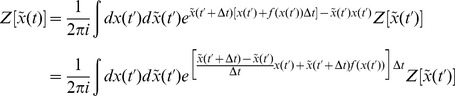
(58)Any given time interval 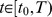 can be divided into 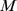 subintervals of length 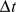. Repeated application of (58) then expresses the generating functional at time 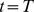 as
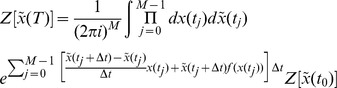
where 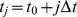. Taking the 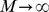 limit gives the functional or path integral
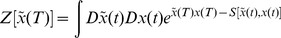
where the measure is defined as
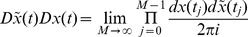
with the 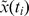 integrations following a contour parallel to the imaginary axis and the 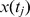 integrations following a contour parallel to the real axis. The action 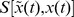 is
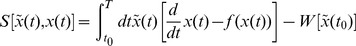
(59)where we have integrated by parts and expressed the initial generating functional in terms of the cumulant generating functional 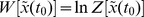. Note that the bracketed term is the left hand side of the differential equation (54). This property is generic and provides a short cut for deriving the action. Because the initial distribution is normalized we have
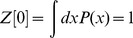
(60)The path integral thus defines a normalized measure when 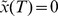. The generating function for the synaptic drive 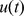 will directly follow this prescription, where the initial probability density 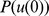 is for similarly prepared networks. The action will have the form of (59), with (2′) replacing the ODE for 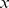.

For the population density 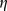, the generating function becomes a generating functional and the expectation value which defines it is a functional integral over the possible values of the field 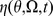. Again we introduce a response field 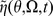 in order to define
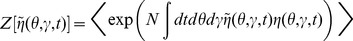
(61)where the expectation value is taken over the ensemble of similarly prepared networks. The experimental preparation of the network is equivalent to choosing the initial network configuration from some ensemble distribution. We will address the exact form of this distribution below, but for now it will suffice to note that this implies an initial generating functional for the initial time 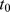. We focus on the time evolution of 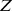 here. The derivation above in terms of the single variable 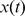 works equally well in the network case (consider the arguments to the field as indices for a configuration vector 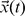). The probability density functional of 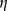 at a 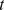 is given by
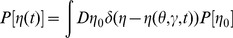
(62)where 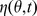 is the solution to the Klimontovich equation (6). This produces the probability density functional
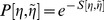
(63)with action
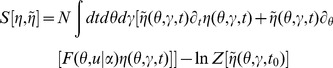
(64)The action completely defines the system and all moments of the ensemble distribution can be computed from it. However, in general, closed form expressions will not be possible and thus perturbation theory is used. The appearance of the factor of 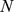 tells us immediately how to calculate finite size corrections to the infinite 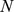 network in terms of a perturbative expansion in 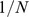 (cf. a steepest descent evaluation of a standard integral with integrand 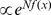 for large 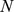).

For the coupled system, we have both the synaptic variable 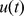 as well as the population density 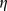. The action for the coupled system is just the sum of the actions for the variables 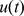 and 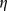.
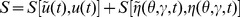
(65)where coupling terms have been included implicitly in the dependence of 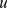 and 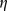 upon each other.

#### Initial distributions

The initial values of the generating functions will be determined by the ensemble distribution of the initial state. In the simplest case, we will consider that the initial state of the synaptic drive variable 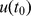 is fixed; furthermore, we will choose it to be fixed at 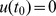. This means that
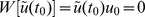
The initial state of the population density 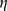 is imposed by the N-neuron distribution of the initial state of the network, 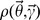 (note that we use the terminology of plasma physics where the ensemble distribution is equivalent to an 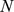 variable joint probability density function). In order to compute the initial state of 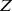, we must compute the following ensemble average over this distribution.
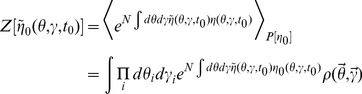
(66)Using the definition of the population density 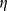 (equation (4)), we can write
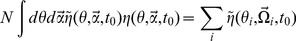
(67)where the index 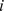 runs over the neurons in the network. This means that the initial generating functional 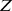 is equivalent to
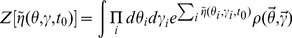
(68)which is the generating function for the ensemble distribution specified by 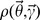. Consider an initial distribution that is independent for each neuron, which means that 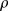 factors into a product over all neurons in the network. Thus
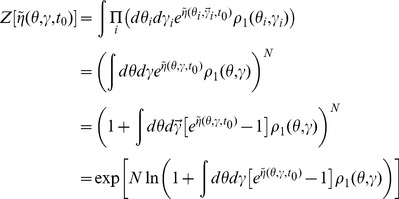
(69)where 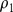 is the one-neuron distribution function marginalized from the N-neuron distribution. We choose the notational convention 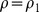. The first term of an expansion of the logarithm in (69) about 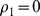 gives precisely the term which would appear in a Poisson distribution. The remaining terms account for the sampling corrections to the 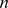-neuron distributions due to a finite number of neurons.

If the neurons are not prepared independently then the expressions for the connected 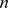-neuron distributions (such as 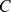) will appear as coefficients of powers of 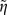 in the exponent along with a combinatoric factor of 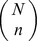, e.g.
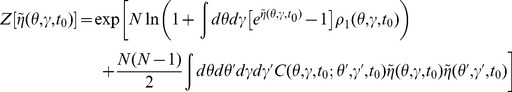
(70)assuming the other connected 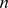-neuron distributions are zero at 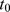.

#### Doi-Peliti-Janssen transformation

Just as the nonlinear terms in the cumulant generating function 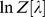 are the “noise” terms, the nonlinear terms in the response fields 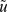 and 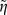 in the actions 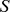 determine the correlations in the fields 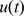 and 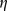. Since the dynamics are deterministic, the initial distribution for the ensemble is the only part of the action which provides non-trivial correlations. This follows because the only introduction of “noise” per se has been through the the fact that the initial conditions and parameters of the network are drawn from a distribution. However, once those are decided, the dynamics are fixed and completely deterministic. It is difficult to compute the effects of fluctuations due to the initial state because the term 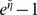 that appears in the initial generating functional. This term is “linearized” by a transformation similar to a Cole-Hopf transformation, which we call the Doi-Peliti-Jannsen transformation [58], given by
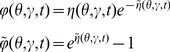
(71)The form of 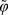 is specified by the Poisson distribution, while the form of 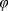 is derived by imposing the requirement that the transformation preserves bilinear derivative forms, i.e.
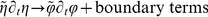
(72)These boundary terms do not contribute to the moments and can be ignored. The Doi-Peliti-Jannsen transformation replaces the Poisson term 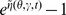 in the action with 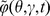. Hence, an action which is bilinear in 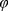, 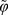 represents a Markov counting process whose solution is a Poisson distribution with mean 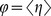.

In a more general case, the Doi-Peliti Janssen transformation provides an elegant means of expanding around Poisson solutions and is thus useful for models whose statistics should be near Poisson, such as population densities in networks, in which the statistics are essentially coupled counting processes, though not simple ones. The moments of the variables 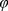 are the joint distributions of 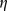 with the finite size sampling corrections removed. We call these moments factorial moments or *normal ordered moments*, borrowing the terminology from the field theory literature. The moments of 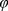 do not include the effects of coincident indices, which is to say they are moments from a distribution without replacement (i.e. there is no probability of drawing the same neuron twice). The distribution implied by the moments of 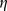 is derived from drawing with replacement.

### Feynman Rules for neural models

The neural models we describe have two different fields, one for the synaptic drive variable and one for the density variable (along with the response field counterparts). As above, the class of models we consider is given by
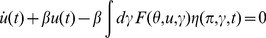
(2′)

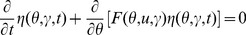
(6)Each term in the expansion of any given moment (such as 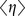 or 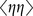) can be represented in an economical fashion via the use of diagrams. The basic elements of these diagrams are completely determined by the action, as derived above. To begin, we expand each action about some solution of mean field theory 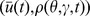, i.e. shift the variables by 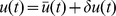, 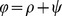. This gives us 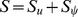, where
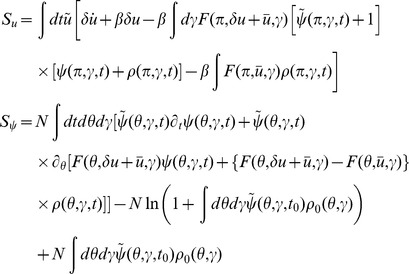
(73)Each term in the action (post expansion) containing anything other than precisely one response field and one configuration field is called a “vertex” term because these terms constrain the types of vertices for our diagrams. The terms with one response field and one configuration field are linear responses and correspond to edges of the graphs. For our models, the linear responses are the solutions of
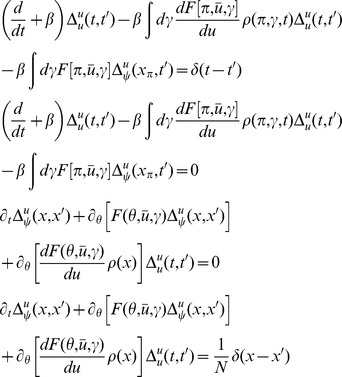
There are four linear response functions, and so four types of edges. Time is considered in diagrams to move from right to left. Edges are represented by a combination of solid and dashed lines. A completely solid line represents the linear response 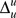, i.e. a response in 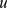 due to a linear perturbation in 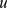. Completely dashed lines represent 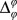, i.e. a response in the density due to perturbations in the density. Mixed edges represent the “off diagonal” linear responses, with the perturbation on the rightward end of the edge and the configuration variable on the left edge.

**Figure 4:**
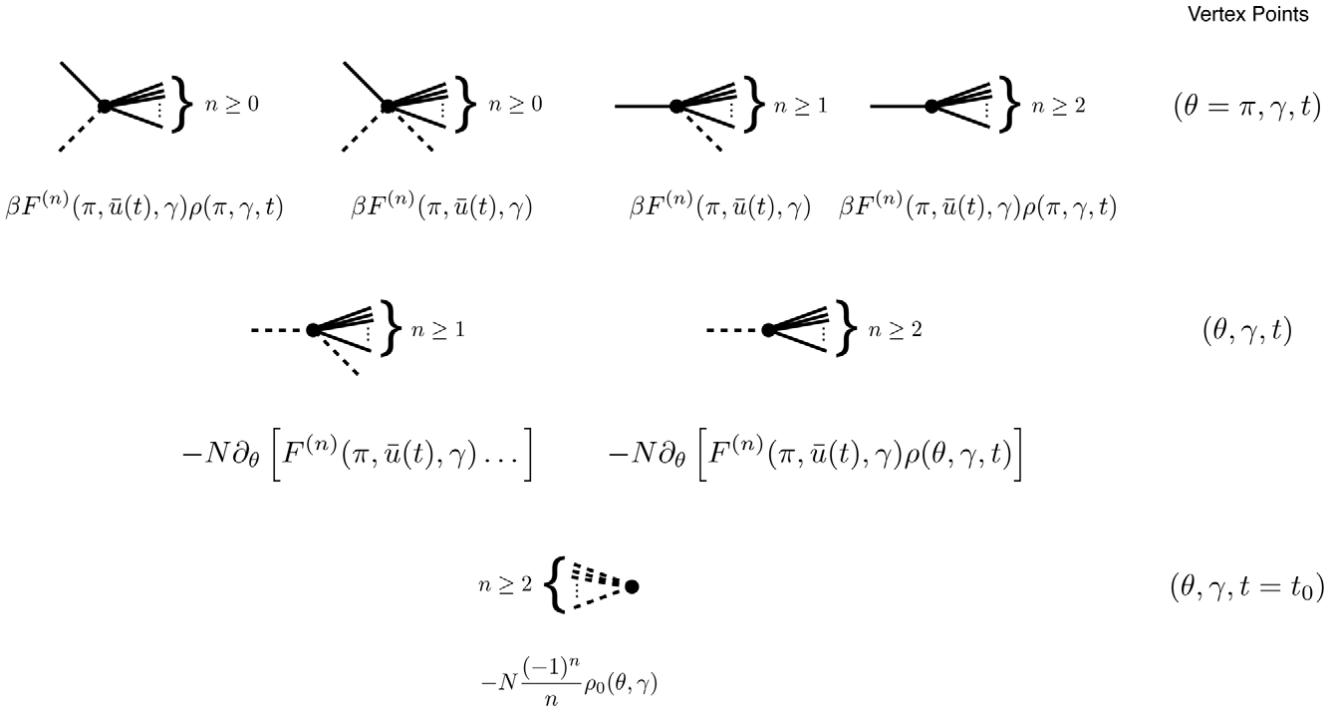
Vertices of the Feynman Rules for the neural models.

Graphs are constructed by connecting the vertices shown in Figure 4 using the four edges defined by the linear response. The terms in the diagrams are constructed by multiplying each vertex factor shown in Figure 4 by the factors of the linear response corresponding to each of the edges and integrating over the open variables indicated by each vertex. Moments are given by the sum of all diagrams with open edges corresponding to the variables in the moment, e.g. the moment 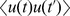 is given by the sum of all graphs with two leftward edges that end in solid lines. Finally, it can be shown [59] that the order of each diagram in 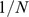 is given by the number of “loops” in the topology of each graph, with higher moments having “tree level” graphs (those with no loops) of order 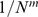, where 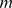 is the order of the moment, i.e. the tree level graphs for 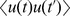 are 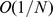.

**Figure 5:**
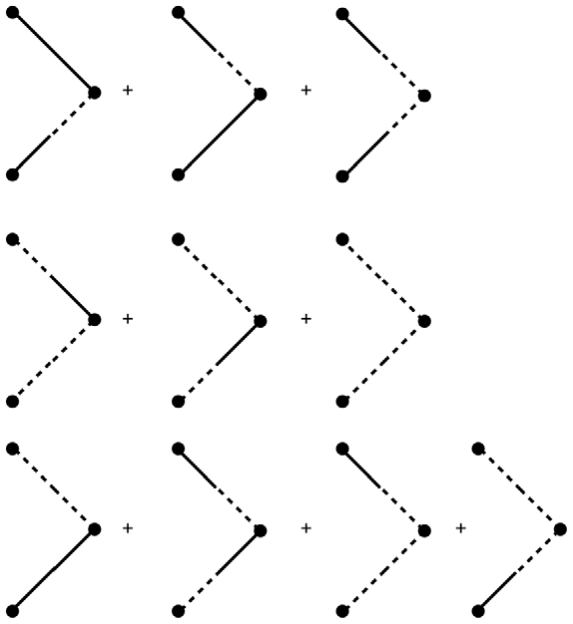
Feynman diagrams for the connected two point correlation functions in the neural field models. By row they are 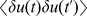, 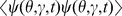, and 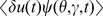.

The graphs for the two point correlations are shown in Figures 5. They correspond to the following terms:
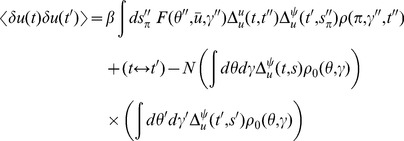
(74)

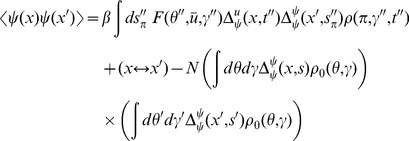
(75)

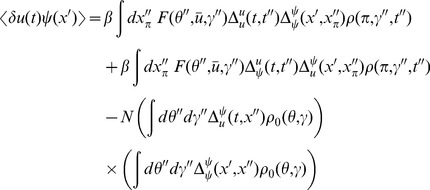
(76)and the variations in the density are given by
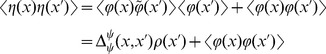
(77)and
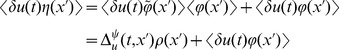
(78)where we've assumed 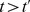. Higher moments can be constructed by considering higher order diagrams.

### Reduction to ODEs

In order to compute the linear response for the quadratic integrate-and-fire model, we use a reduction to a simple system of ODEs. We start with the propagators in steady state in the 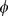 representation:
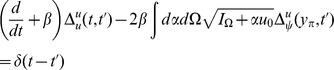


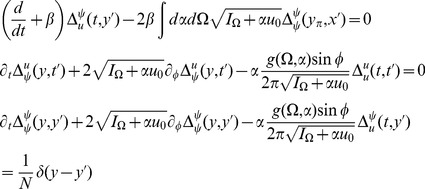
(79)Define
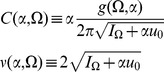
(80)Then we have
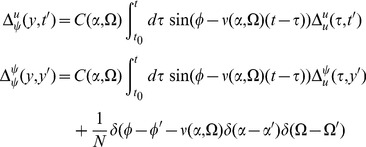
(81)We are interested in solving for the value at 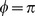. Define
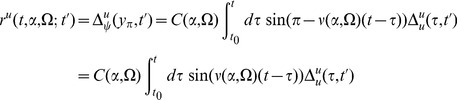
(82)where 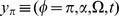. Also define
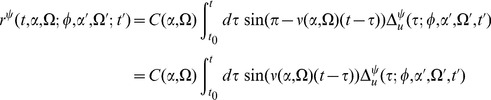
(83)The equations for 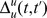 and 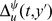 are
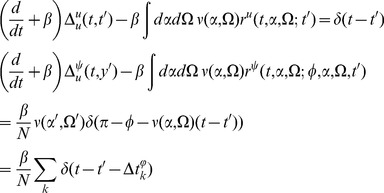
(84)where 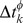 are defined such that 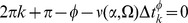.

Let's derive equations for the 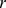
's. Taking the time derivative gives us
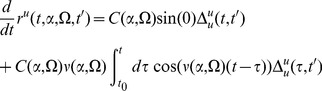
(85)a second derivative gets us
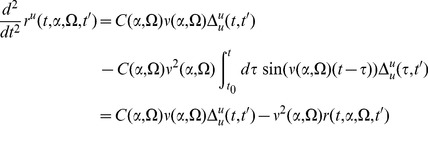
(86)So the pair of propagators involving 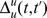 is given by
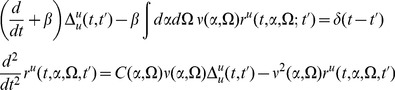
(87)The same procedure works for the other pair to give us
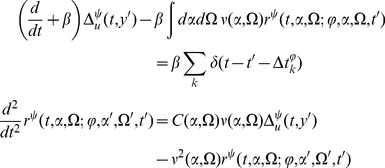
(88)


#### Propagators convolved with initial conditions

We start with 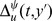. Convolving the relevant pair of propagators with the initial (steady state) density 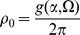 gives us
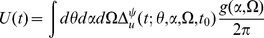
(89)This reduces to the same set of equations as for 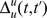 with the addition of a constant driving term.
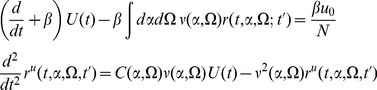
(90)All of the reduced equations were solved numerically with the midpoint method.
